# Correction to ‘Modelling stem cell ageing: a multi-compartment continuum approach’

**DOI:** 10.1098/rsos.200836

**Published:** 2020-06-03

**Authors:** Yanli Wang, Wing-Cheong Lo, Ching-Shan Chou

*R. Soc. Open Sci.* **7**, 191848. (Published online 18 March 2020) (doi:10.1098/rsos.191848)

This correction refers to an error in the spelling of one of the authors' names. Ching-Shin Chou was given, in place of Ching-Shan Chou. This has now been corrected.

